# Annual and seasonal patterns in etiologies of pediatric community-acquired pneumonia due to respiratory viruses and *Mycoplasma pneumoniae* requiring hospitalization in South Korea

**DOI:** 10.1186/s12879-020-4810-9

**Published:** 2020-02-12

**Authors:** Eun Lee, Chul-Hong Kim, Yong Ju Lee, Hyo-Bin Kim, Bong-Seong Kim, Hyung Young Kim, Yunsun Kim, Sangyoung Kim, Chorong Park, Ju-Hee Seo, In Suk Sol, Myongsoon Sung, Min Seob Song, Dae Jin Song, Young Min Ahn, Hea Lin Oh, Jinho Yu, Sungsu Jung, Kyung Suk Lee, Ju Suk Lee, Gwang Cheon Jang, Yoon-Young Jang, Eun Hee Chung, Hai Lee Chung, Sung-Min Choi, Yun Jung Choi, Man Yong Han, Jung Yeon Shim, Jin Tack Kim, Chang-Keun Kim, Hyeon-Jong Yang, Pneumonia and Respiratory Disease Study Group of Korean Academy of Pediatric Allergy and Respiratory Disease

**Affiliations:** 1 Department of Pediatrics, Chonnam National University Hospital, Chonnam National University Medical School, Gwangju, South Korea; 2 Department of Pediatrics, Samsung Changwon Hospital, Sungkyunkwan University School of Medicine, Changwon, South Korea; 3 Department of Pediatrics, Hallym University Kangnam Sacred Heart Hospital, Seoul, South Korea; 4 Asthma and Allergy Center, Department of Pediatrics, Inje University Sanggye Paik Hospital, 1342 Dongil-ro, Nowon-gu, Seoul, 01757 South Korea; 5 Department of Pediatrics, Ulsan University Gangneung Asan Hospital, Gangneung, South Korea; 6 Department of Pediatrics, Pusan National University Children’s Hospital, Yangsan, South Korea; 7 SCH Biomedical Informatics Research Unit, Soonchunhyang University Seoul Hospital, Seoul, South Korea; 8 Department of Pediatrics, Dankook University Hospital, Dankook University Medical School, Cheonan, South Korea; 9 Department of Pediatrics, Hallym University Chuncheon Sacred Heart Hospital, Chuncheon, South Korea; 10 Department of Pediatrics, Soonchunhyang University Gumi Hospital, Seoul, South Korea; 11 Department of Pediatrics, Inje University Haeundae Paik Hospital, Pusan, South Korea; 12 Department of Pediatrics, Korea University Guro Hospital, Korea University College of Medicine, Seoul, South Korea; 13 Department of Pediatrics, Eulji University, Eulji General Hospital, Seoul, South Korea; 14 Department of Pediatrics, Korea Cancer Center Hospital, Seoul, South Korea; 15 Department of Pediatrics, Asan Medical Center, Ulsan University Medical School, Seoul, South Korea; 16 Department of Pediatrics, Hanyang University Guri Hospital, Hanyang University College of Medicine, Guri, South Korea; 17 Department of Pediatrics, National Health Insurance Service, Ilsan Hospital, Ilsan, South Korea; 18 Department of Pediatrics, Catholic University of Daegu School of Medicine, Daegu, South Korea; 19 Department of Pediatrics, Chungnam National University Hospital, Chungnam National University School of Medicine, Daejeon, South Korea; 20 Department of Pediatrics, Dongguk University Kyungju Hospital, Kyungju, South Korea; 21 Department of Pediatrics, Seoul National University Children Hospital, Seoul, South Korea; 22 Department of Pediatrics, CHA Bundang Medical Center, CHA University School of Medicine, Seongnam, South Korea; 23 Department of Pediatrics, Kangbuk Samsung Hospital, Sungkyunkwan University School of Medicine, Seoul, South Korea; 24 Department of Pediatrics, College of Medicine, The Catholic University of Korea, Seoul, South Korea; 25 Department of Pediatrics, Soonchunhyang University Seoul Hospital, Soonchunhyang University College of Medicine, 59 Daesagwan-ro, Yongsan-gu, Seoul, 04401 South Korea

**Keywords:** Children, Pneumonia, Respiratory virus, *Mycoplasma pneumoniae*, Macrolide- refractory, Macrolide-sensitive, Macrolide less-effective

## Abstract

**Background:**

Community–acquired pneumonia (CAP) is one of the leading worldwide causes of childhood morbidity and mortality. Its disease burden varies by age and etiology and is time dependent. We aimed to investigate the annual and seasonal patterns in etiologies of pediatric CAP requiring hospitalization.

**Methods:**

We conducted a retrospective study in 30,994 children (aged 0–18 years) with CAP between 2010 and 2015 at 23 nationwide hospitals in South Korea. *Mycoplasma pneumoniae* (MP) pneumonia was clinically classified as macrolide-sensitive MP, macrolide-less effective MP (MLEP), and macrolide-refractory MP (MRMP) based on fever duration after initiation of macrolide treatment, regardless of the results of in vitro macrolide sensitivity tests.

**Results:**

MP and respiratory syncytial virus (RSV) were the two most commonly identified pathogens of CAP. With the two epidemics of MP pneumonia (2011 and 2015), the rates of clinical MLEP and MRMP pneumonia showed increasing trends of 36.4% of the total MP pneumonia. In children < 2 years of age, RSV (34.0%) was the most common cause of CAP, followed by MP (9.4%); however, MP was the most common cause of CAP in children aged 2–18 years of age (45.3%). Systemic corticosteroid was most commonly administered for MP pneumonia. The rate of hospitalization in intensive care units was the highest for RSV pneumonia, and ventilator care was most commonly needed in cases of adenovirus pneumonia.

**Conclusions:**

The present study provides fundamental data to establish public health policies to decrease the disease burden due to CAP and improve pediatric health.

## Background

Lower respiratory tract infections, including pneumonia, are a group of diseases that constitute a leading worldwide cause of pediatric morbidity, requiring hospitalization, and mortality [1, 2]. Childhood pneumonia causes diverse long-term sequelae, such as restrictive or obstructive lung disease, as well as bronchiectasis, particularly in a considerable proportion of children hospitalized due to community-acquired pneumonia (CAP) [3].

The etiologies of CAP affect the disease burden, development of long-term sequelae, and mortality [3, 4]. The mortality, severity, and disease burden due to CAP differ by age [3, 5]. Therefore, prediction of the causative pathogens and clinical courses before the identification of respiratory pathogens in children with CAP is needed to improve disease outcomes. There are annual and seasonal variabilities in the respiratory pathogens that cause CAP. In addition, the characteristics of these pathogens, including susceptibility to antibiotics, change over time. Assessments of the annual and seasonal variabilities and pathogen characteristics could provide important information needed to establish a direction for vaccine development and health care policies. However, comprehensive studies on these topics are scarce.

The most common causes of pediatric CAP in high-income countries, which differ according to age, are *Mycoplasma pneumoniae* (MP) and respiratory viruses [6]. Epidemics of MP infections are repeated on a 3–7 year cycle; the last epidemic in Korea was in 2015 [7–9]. After 2000, macrolide-resistant MP pneumonia rapidly emerged, especially in Asia [7]. MP infections in children are often self-limiting, even in patients with macrolide-resistant MP pneumonia [10]. More than 80% of patients with macrolide-sensitive MP (MSMP) have shown defervescence within 48–72 h after the initiation of macrolide treatment; this occurs in approximately 30% of patients with macrolide-resistant MP pneumonia [11]. Despite its high prevalence, large-scale epidemiologic studies of macrolide-resistant MP pneumonia are scarce.

In the present study, we aimed to identify the annual and seasonal patterns of respiratory pathogens in pediatric CAP requiring hospitalization and the prevalence of MP pneumonia according to the clinical responses to macrolides. In addition, we compared the clinical characteristics according to respiratory pathogens in patients with pediatric CAP.

## Methods

### Study population

We conducted a retrospective chart review of children hospitalized with CAP between January 2010 and December 2015 in a cohort of children younger than 18 years of age from 23 medical centers in South Korea. During the study period, a total of 65,243 children were diagnosed with CAP based on clinical features, chest radiography, and laboratory findings. Among them, 30,994 children underwent real-time polymerase chain reaction (RT-PCR) analyses for respiratory virus (*n* = 30,994) or MP (*n* = 9197), or underwent serology tests for MP (*n* = 30,994), to identify the etiologies of CAP [7]. To identify differences in causative pathogens according to age, the enrolled children were divided into four age groups as follows: < 2 years, 2 years to < 5 years, 5 years to < 12 years, and 12 years to < 18 years. Information regarding the clinical characteristics, radiologic findings, and laboratory findings was collected from the patient’s electronic medical record. The Institutional Review Boards of all participating medical centers reviewed and approved the study protocol.

### Definition

Pneumonia was diagnosed by pediatricians based on both physical examinations and radiologic assessments. MP pneumonia was defined as meeting at least one of the following criteria: (1) positive results in both RT-PCR analysis of nasopharyngeal samples and the specific IgM against MP at the time of hospitalization due to CAP, (2) seroconversion of the specific IgM against MP, or (3) four-fold or greater increase in the specific IgG against MP in the acute and convalescent stages [8, 12].

The clinical courses of MP pneumonia have shown heterogeneous features, even in patients with the same in vitro sensitivity to macrolide results [8, 9, 13]. Some previous studies have shown no significant differences in clinical, laboratory, and radiologic features between children with macrolide-resistant MP pneumonia and those with MSMP pneumonia [7–9]. In the present study, MP susceptibility tests for macrolides were not performed. Instead, to characterize the clinically heterogeneous MP pneumonia into more homogenous clinical groups, we defined clinical MSMP, macrolide-refractory MP (MRMP), and macrolide less-effective MP (MLEP) pneumonia according to fever duration in each pneumonia episode after the initiation of macrolides, regardless of the sensitivity test for macrolides, as follows: fever for less than 3 days, fever for more than 7 days, and more than 3 days but less than 7 days, respectively.

### Microbiological studies

RT-PCR was performed to identify the causative respiratory viruses using an Anyplex II RV 16 Detection kit (Seegene, Seoul, Korea) [8]. The identified respiratory viruses were as follows: adenovirus (AdV), human rhinovirus (HRV), influenza virus (FLU), parainfluenza virus (PIV), human metapneumovirus (HMPV), respiratory syncytial virus (RSV), bocavirus (BoV), and human coronavirus (HCoV). RT-PCR was performed to identify MP using an ABI 7500 (Applied Biosystems, Foster City, CA, USA) with an EuDxTM-PN MLC Detection Kit (EUDIPIA) and an AnyplexTM II RB5 Detection Kit (Seegene). The results of RT-PCR analyses and serologic titers for MP infections were obtained from comprehensive reviews of laboratory records.

### Statistical analysis

Seasonal Mann-Kendall tests, one type of non-parametric statistical analysis, were used to detect monotonic trends in monthly data with an annual seasonal pattern. For the categorical variables, numbers and percentages were calculated and groups were analyzed using the chi-squared test. Post hoc analyses were used to examine if the characteristics of MP pneumonia groups were statistically significant with the chi-squared test. For the parametric continuous variables, means and standard deviations (SD) were derived, and one-way ANOVA tests were conducted to compare groups, followed by Bonferroni’s post hoc test. For the nonparametric continuous variables, the Kruskal-Wallis rank test was performed, followed by Dunn’s post hoc test. All statistical analyses were performed using R 3.5.1. A *p* value of < 0.05 was considered statistically significant.

## Results

### Characteristics of the study population

A total of 30,994 children with CAP requiring hospitalization were enrolled in the present study. The characteristics of the study population are presented in Table 1. The mean age of the participants was 41.9 months (SD, 40.8 months) and 54.6% of the total study population were boys. The number of children hospitalized due to CAP was highest in fall (September to November), followed by winter (December to February), spring (March to May), and summer (June to August).

**Table 1:** Demographic characteristics of hospitalized children with community-acquired pneumonia

Variables	No. (%) or mean ± SD
Number	30,994
Age (mo), mean ± SD	41.9 ± 40.8
< 2 yrs	13,527 (43.6)
2 yrs. ≤ age < 5 yrs	9715 (31.3)
5 yrs. ≤ age < 12 yrs	5802 (18.7)
12 yrs. ≤ age < 18 yrs	1950 (6.3)
Sex (male), No. (%)	16,929 (54.6)
Incident year, No. (%)	30,991 (100.0)
2010	4001 (12.9)
2011	6427 (20.7)
2012	3753 (12.1)
2013	2913 (9.4)
2014	5702 (18.4)
2015	8195 (26.44)
Incident season	30,994 (100.0)
Spring (Mar, Apr, May)	7765 (25.1)
Summer (Jun, Jul, Aug)	5124 (16.5)
Fall (Sep, Oct, Nov)	10,065 (32.5)
Winter (Dec, Jan, Feb)	8040 (25.9)

*Mo* months, *n* number, *SD* standard deviation, *yrs* years

### Annual and seasonal patterns of CAP caused by respiratory viruses and MP

**Fig. 1:**
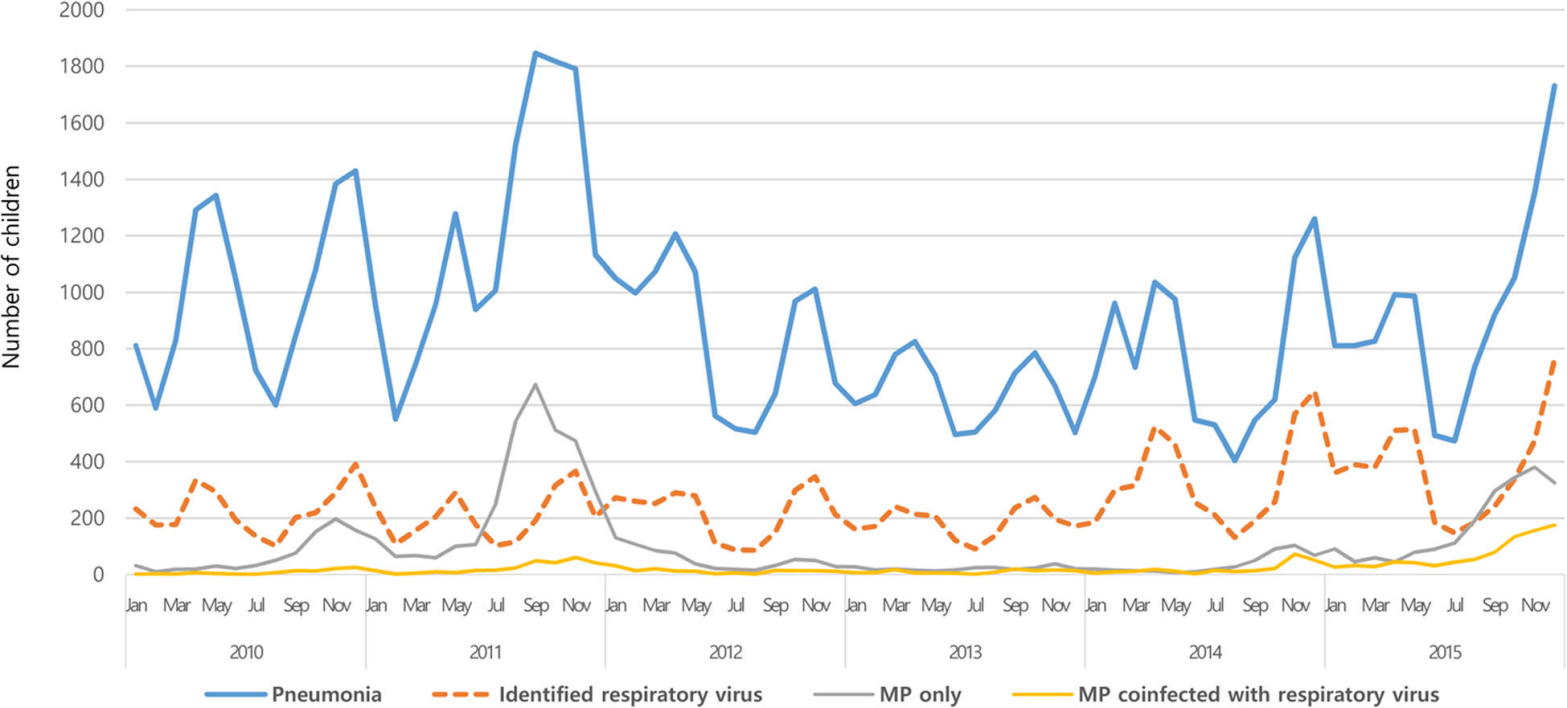
Number of children hospitalized with community-acquired pneumonia according to etiology between 2011 and 2015 in Korea

**Fig. 2:**
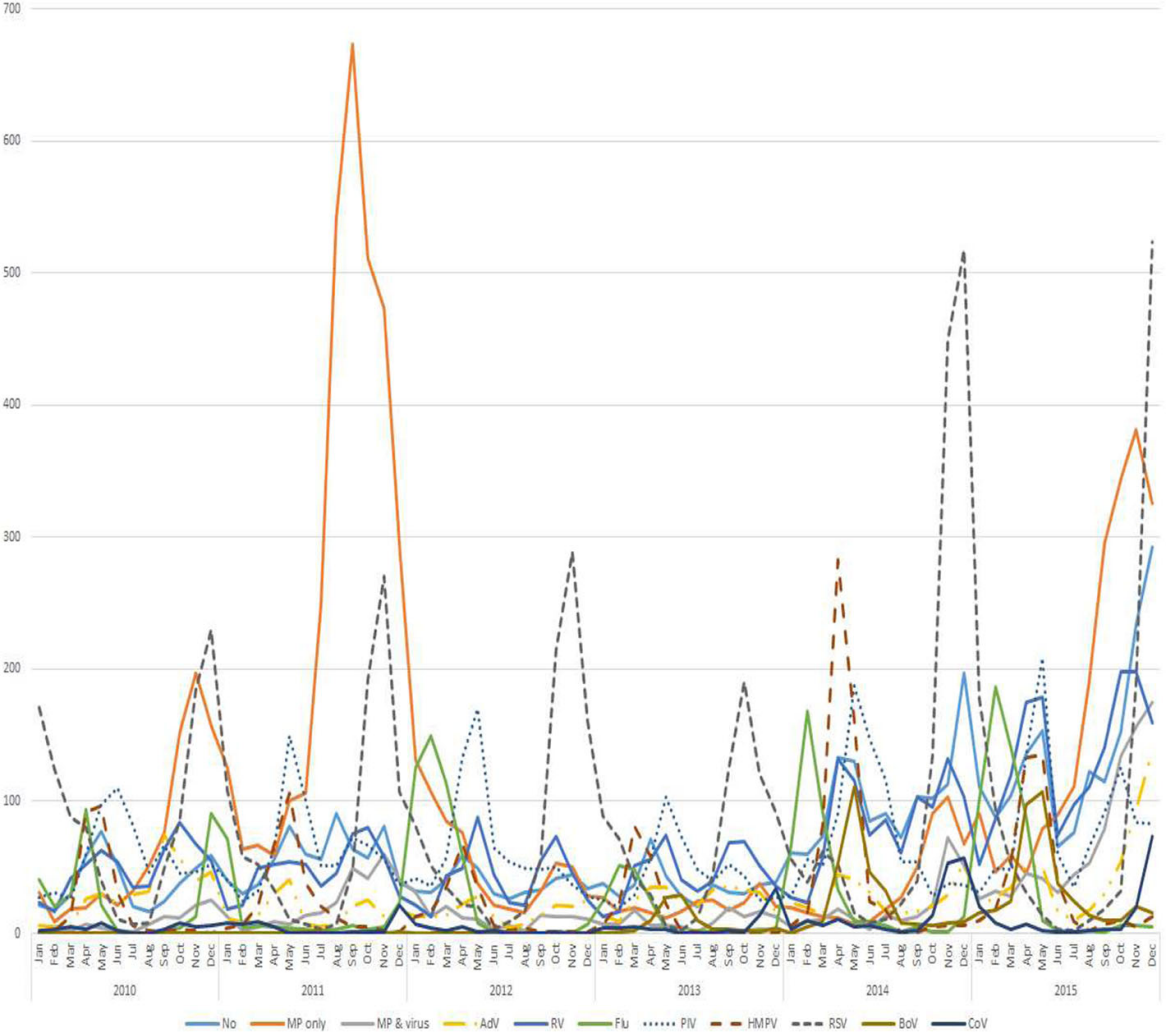
Number of children hospitalized with community acquired pneumonia according to etiology

During the study period, the number of children hospitalized due to CAP was highest in 2011, followed by 2015 (Fig. 1). The number of children hospitalized with CAP due to various respiratory virus infections was highest in 2015, with similar seasonal and annual patterns of total CAP. Among the various respiratory viruses, RSV was the most commonly identified virus, especially from November to December, with a gradually increasing trend until each peak (Fig. 2). The number of children hospitalized due to FLU-associated CAP peaked from January to March in each year, with the largest number of affected patients in 2015. The number of children hospitalized with HMPV-associated CAP peaked in April, with its largest number of affected patients in 2014 (Additional file 1: Figure S1).

**Fig. 3:**
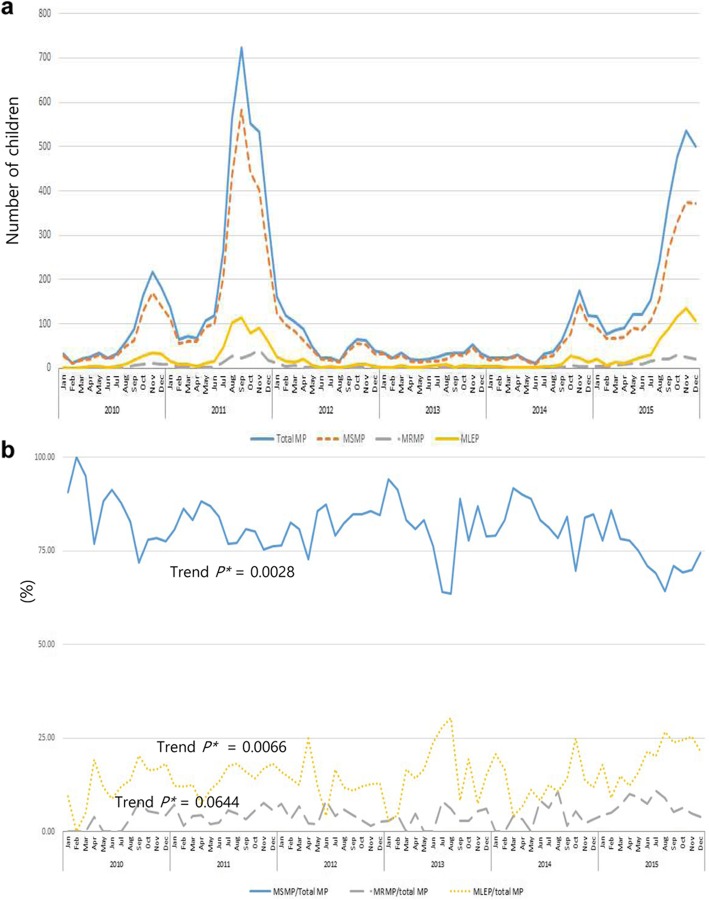
Time-dependent changes in the numbers of children hospitalized due to *Mycoplasma pneumoniae* (MP) pneumonia according to clinical macrolide sensitivity. **a** Number of children hospitalized with MP pneumonia classified as macrolide-sensitive (MSMP), macrolide-refractory (MRMP), and macrolide less-effective (MLEP). **b** Time-dependent changes in the ratios of MSMP/total MP pneumonia, MRMP pneumonia/total MP pneumonia, and MLEP pneumonia/total MP pneumonia during the study period

The two epidemics of MP pneumonia (2011 and 2015) corresponded with the two peaks of CAP. The incidence of MP pneumonia requiring hospitalization in children peaked from October to November in each year (Fig. 1). The most common clinical phenotype of MP pneumonia was MSMP, followed by MLEP and MRMP (Fig. 3a). During the study period, the monthly rate of MSMP/total MP pneumonia was 63.6–100.0% (mean ± SD, 81.3 ± 7.7), that of MRMP/total MP pneumonia was 0.0–11.0% (4.2 ± 4.3), and that of MLEP/total MP pneumonia was 0.0–30.3% (14.5 ± 14.2) (Fig. 3b). The rates of clinical MLEP and MRMP pneumonia showed increasing trends (seasonal Mann-Kendall trend tests, *P* = 0.0644 and *P* = 0.0066, respectively), whereas the rates of clinical MSMP pneumonia showed a significant reduction during the study period (seasonal Mann-Kendall trend test, *P* = 0.0028).

### Causative pathogens of CAP according to age

The number of children hospitalized with CAP decreased with age (Table 2). In children younger than 2 years of age, RSV (34.0%) was the most commonly detected respiratory pathogen, followed by PIV (19.7%), and HRV (18.8%). In children 2–4 years of age, RSV (14.9%) was the most commonly identified respiratory virus, followed by HRV (14.8%), and PIV (13.6%). In children 5–17 years of age, HRV (10.6%) was the most commonly detected virus, followed by FLU (9.2%). In children older than 2 years of age, MP (45.3%) was the most common cause of CAP, whereas 9.4% of children younger than 2 years of age exhibited MP infections. The rate of clinical MRMP/total MP pneumonia was highest in children 5–9 years of age (244/3475, 9.8%), followed by adolescents 10–17 years of age (47/905, 6.9%), children 2–4 years of age (154/3526, 4.4%) and those younger than 2 years of age (25/1277, 2.0%).

**Table 2:** Prevalence of causative respiratory viruses and *Mycoplasma pneumoniae* according to age groups in children hospitalized due to community-acquired pneumonia

Detected respiratory virus	< 2 years, No. (%)	2–4 years, No. (%)	5–11 years, No. (%)	12–17 years, No. (%)	Total, No. (%)
Adenovirus	964 (7.1)	764 (7.9)	183 (3.12)	33 (1.7)	1944 (6.3)
Rhinovirus	2545 (18.8)	1441 (14.8)	626 (10.8)	199 (10.2)	4811 (15.5)
Influenza	701 (5.2)	619 (6.44)	519 (9.0)	196 (10.1)	2035 (6.6)
Influenza A	534 (4.0)	386 (4.0)	302 (5.2)	117 (6.0)	1339 (4.32)
Influenza B	172 (1.3)	239 (2.5)	218 (3.8)	79 (4.1)	708 (2.3)
Parainfluenza	2668 (19.7)	1317 (13.6)	476 (8.2)	192 (9.9)	4653 (15.0)
Metapneumovirus	1049 (7.8)	750 (7.7)	112 (1.9)	45 (2.3)	1956 (6.3)
Respiratory syncytial virus	4600 (34.0)	1451 (14.9)	194 (3.3)	59 (3.0)	6304 (20.3)
Bocavirus	511 (38.0)	214 (2.2)	41 (0.7)	20 (1.0)	786 (2.5)
Coronavirus	264 (2.0)	152 (1.6)	66 (1.1	21 (1.1)	503 (1.6)
MP	1277 (9.4)	3526 (36.3)	3475 (59.9)	905 (46.4)	9183 (29.6)
Clinically MSMP	1121 (8.3)	2816 (29.0)	2482 (42.8)	681 (34.9)	7100 (22.9)
Clinically MRMP	25 (0.2)	154 (1.6)	244 (4.2)	47 (2.4)	470 (1.5)
Clinically MLEP	131 (1.1)	556 (5.7)	749 (12.9)	177 (9.1)	1613 (5.2)
Neither MP nor virus identified	1997 (14.8)	1529 (15.7)	906 (15.6)	484 (24.8)	4916 (15.9)
Only respiratory virus detection	10,253 (75.8)	4660 (48.0)	1421 (24.5)	561 (28.8)	16,895 (54.5)
Total number	13,527 (100.0)	9715 (100.0)	5802 (100.0)	1950 (100.0)	30,994 (100.0)

*MLEP* macrolide less-effective *Mycoplasma pneumoniae*, *MP Mycoplasma pneumoniae*, *MRMP* macrolide-refractory *Mycoplasma pneumoniae*, *MSMP* macrolide-sensitive *Mycoplasma pneumoniae*, *No* Number

### Clinical characteristics and laboratory findings according to etiologies of CAP

The clinical features and laboratory findings, according to the etiologies, in children admitted due to CAP are described in Table 3. The median length of hospital stay due to CAP was 181.39 h. The duration of hospitalization due to CAP was longest in those with AdV pneumonia, followed by RSV pneumonia. Systemic corticosteroids were most commonly administered to those with MP pneumonia (1645/7455, 22.1%), followed by RSV pneumonia (622/4521, 13.8%). The number of children with CAP who required oxygen supplementation was greatest in children with RSV pneumonia (905/4521, 20.0%); ventilator care was most commonly applied in children with AdV pneumonia (13/680, 1.9%), followed by those with RSV pneumonia (119/4521, 2.6%).

**Table 3:** Comparison of clinical and laboratory findings according to respiratory pathogens in children hospitalized due to community-acquired pneumonia

Variables	only MP^1^ (*n* = 7455)	MP coinfected with virus^2^ (*n* = 1728)	Adenovirus^3^ (*n* = 680)	Influenza virus^4^ (*n* = 1491)	Metapneumovirus^5^ (*n* = 1287)	RSV^6^ (*n* = 4521)	*P* value	Post hoc test^a^
Age (month), mean ± SD	66.7 ± 39.7	53.3 ± 37.3	34.1 ± 28.0	54.4 ± 46.0	28.1 ± 28.2	15.9 ± 21.0	< 0.0001	1 > 4 = 2 > 3 > 5 > 6
Sex (male), No. (%)	3680 (49.4)	862 (49.9)	389 (57.2)	828 (55.5)	692 (53.8)	2551 (56.4)	< 0.0001	2 > 3 = 6 = 4 = 5 > 1
Duration of Hospitalization (hrs), mean ± SD	159.9 ± 227.4	174.2 ± 339.8	181.4 ± 250.4	136.3 ± 161.2	148.6 ± 166.0	162.2 ± 238.6	< 0.0001	3 = 2 = 6 = 1 ≥ 5 ≥ 4
Administration of steroid, No. (%)	1645 (22.1)	454 (26.3)	75 (11.0)	117 (7.9)	170 (13.2)	622 (13.8)	< 0.0001	2 > 1 > 6 = 5 = 3 > 4
Oxygen supplementation, No. (%)	299 (4.0)	109 (6.3)	67 (9.9)	82 (5.5)	147 (11.4)	905 (20.0)	< 0.0001	6 > 5 > 3 > 2 = 4 = 1
Ventilator care, No. (%)	12 (0.2)	4 (0.2)	8 (1.2)	14 (0.9)	12 (0.9)	51 (1.1)	< 0.0001	3 = 6 > 4 = 5 = 2 = 1
ICU care, No. (%)	27 (0.4)	11 (0.6)	13 (1.9)	23 (1.5)	17 (1.3)	119 (2.6)	< 0.0001	6 ≥ 3 = 4 = 5 = 2 > 1
WBC (× 10^3^/μL), median ± IQR	8275 ± 4700	8870 ± 5560	11,810 ± 7155	7510 ± 4760	8700 ± 5142.5	9660 ± 4857.5	< 0.0001	3 > 2 > 4 > 6 > 1 = 5
Neutrophil (%), median ± IQR	60.1 ± 21.8	57.6 ± 26.2	56.2 ± 25.8	57.5 ± 32.2	42.3 ± 29.6	35 ± 28.4	< 0.0001	1 > 2 = 3 = 4 > 5 > 6
Lymphocyte (%), median ± IQR	28.5 ± 18.8	31 ± 22.8	33 ± 25	29.7 ± 28	46 ± 27.2	51.8 ± 26.5	< 0.0001	6 > 5 > 2 = 3 = 4 > 1
Eosinophil (%), median ± IQR	1.6 ± 3.3	1.1 ± 2.5	0.4 ± 1.4	0.4 ± 1	0.5 ± 1.2	1 ± 2	< 0.0001	1 > 2 > 6 > 3 = 4 = 5
Hb, median ± IQR	12.4 ± 1.2	12.3 ± 1.2	11.8 ± 1.2	12.3 ± 1.5	12 ± 1.3	11.9 ± 1.7	< 0.0001	1 ≥ 2 = 4 > 5 ≥ 6 ≥ 3
Platelet (×10^3^/μL), median ± IQR	293.0 ± 140.0	284.0 ± 126.0	297.0 ± 144.8	232.0 ± 102.0	260.0 ± 132.0	328.0 ± 166.0	< 0.0001	6 > 1 ≥ 3 ≥ 2 > 5 > 4
AST, U/L, median ± IQR	29.0 ± 9.0	30.0 ± 9.0	31.0 ± 8.0	31.0 ± 9.0	34.0 ± 8.0	33.0 ± 8.0	< 0.0001	5 ≥ 6 > 3 = 4 = 2 > 1
ALT, U/L, median ± IQR	14.0 ± 7.0	14.0 ± 7.0	14.0 ± 8.0	15.0 ± 9.0	16.0 ± 9.0	18.0 ± 10.0	< 0.0001	6 > 5 = 4 > 3 = 2 = 1
LDH, U/L, median ± IQR	479.0 ± 294.5	475.0 ± 348.5	516.5 ± 326.8	467.0 ± 292.5	549.0 ± 311.0	540 ± 300	< 0.0001	6 > 5 = 3 = 2 = 1 > 4
CRP, mg/dL, median ± IQR	2.2 ± 4.8	1.6 ± 3.8	2.7 ± 4.9	1.0 ± 2.6	1.1 ± 2.8	0.6 ± 1.8	< 0.0001	1 ≥ 3 ≥ 2 > 5 = 4 > 6
ESR, mm/hr., median ± IQR	31.0 ± 30.0	30.0 ± 30.0	34.0 ± 35.0	14.0 ± 19.2	20 ± 23	15.0 ± 22.0	< 0.0001	3 > 1 = 2 > 5 > 6 > 4

*ALT* alanine aminotransferase, *AST* aspartate aminotransferase, *CRP* C-reactive protein, *ESR* erythrocyte sedimentation rate, *Hb* hemoglobin, *ICU* intensive care unit, *LDH* lactate dehydrogenase, *MP Mycoplasma pneumoniae*, *NA* not applicable, *RSV* respiratory syncytial virus, *SD* standard deviation, *WBC* white blood cell count

^a^Group 1, MP pneumonia; group 2, MP co-infected with virus; group 3, adenovirus; group 4, influenza virus; group 5, Metapneumovirus; group 6, RSV; The inequality or equal signs mean the rank order of post hoc analyses

^b^Post hoc analyses were used to examine if the characteristics of MP pneumonia groups were statistically significant with chi-squared test. For the continuous variables, one-way ANOVA tests were conducted to compare groups, followed by Mann-Whitney U test

The white blood cell counts were highest in AdV, followed by RSV, HMPV, MP and FLU. Blood neutrophil percentages were highest in MP, followed by FLU, AdV, HMPV, and RSV. C-reactive protein levels were highest in AdV, followed by MP, HMPV, FLU, and RSV.

## Discussion

We evaluated the annual and seasonal patterns in respiratory etiologies of pediatric CAP requiring hospitalization between 2010 to 2015 in a nationwide retrospective cohort study. The most common causes of hospitalization due to pediatric CAP were MP and RSV, with peaks in October–November and November–December, respectively. There were two epidemics of MP pneumonia (2011 and 2015) during the study period. In children hospitalized with CAP due to MP pneumonia, the monthly rates of clinical MRMP and MLEP pneumonias showed increasing trends, together comprising up to 36% of the total cases of MP pneumonia during the study period. In children less than 2 years of age, RSV was the most common cause of pediatric CAP requiring hospitalization, whereas the most common cause was MP in children older than 2 years as well as in adolescents. The rate of children admitted to the intensive care unit was highest in children with RSV pneumonia, followed by those with AdV pneumonia. Ventilator care was most commonly needed in children with AdV pneumonia, followed by those with RSV pneumonia. The results of the present study provide fundamental data on the periodicity of epidemics of pathogens that cause pediatric CAP requiring hospitalization.

In the present study, we found that RSV was the most common cause of CAP in children younger than 2 years of age, which is consistent with the findings of other studies performed in other countries, regardless of the detection methods used or national income levels of the children who were analyzed [6, 14]. The immune response in RSV infection differs according to age [15]. Notably, inefficient and ineffective immune responses in early life contribute to more severe clinical courses and higher incidence of RSV infection; this is especially problematic in infants, who are most frequently affected by RSV pneumonia [15]. These findings may be valuable to guide therapeutic approaches, such as application of immune-modulatory drugs for enhancement of immune responses, and preventive strategies for children with CAP; moreover, the results of the present study suggest that the development of strategies to prevent RSV infection, especially in infants and younger children, might aid in decreasing the worldwide disease burden due to CAP.

The number of children hospitalized with MP pneumonia was typically highest between October and November. In the previous studies, the peak incidence of MP pneumonia showed a similar pattern to that observed in the present study, regardless of age [14, 16]. However, in the 2011 Korean epidemic, the peak rate of hospitalization due to MP pneumonia in children occurred in September, whereas the peak rate in the 2015 epidemic occurred in November. In addition, the number of children hospitalized with MP pneumonia in the 2015 epidemic was smaller than that in the 2011 epidemic. These findings were consistent with the results of another study [16]. The reasons for these phenomenon may be associated with exposure to MP in the previous epidemic [7].

Based on in vitro macrolide sensitivity tests, the macrolide resistance rates of MP pneumonia have recently been identified as 50–90% in Asia [7, 8]; these rates differ dramatically among nations. When we defined clinical MSMP, MLEP, and MRMP according to fever duration after the initiation of macrolides in each pneumonia episode, regardless of the results of in vitro macrolide sensitivity tests, the ratios of clinical MLEP and MRMP of total MP pneumonia showed increasing trends after adjustment for monthly time series. However, the estimated clinical MRMP/total MP pneumonia ratio during the study period in the present study (0.0–11.0%) was far lower than that based on in vitro macrolide sensitivity tests (50–90%, especially in Asia) [7, 8]. Therefore, the results of our present study suggest that the clinical response to macrolides in MP pneumonia in the real world might not be as weak as that has been reported, based on in vitro macrolide sensitivity tests, which could partially be due to the self-limiting features of MP infections [17]. Due to the high prevalence of MRMP, there are a great deal of concerns regarding second-line treatment options for MRMP, including tetracycline or fluoroquinolones [7]. Some previous studies have reported no significant differences with respect to clinical and radiologic findings between MRMP and MSMP pneumonia in children [8, 9]. When combined with the results of the present and previous studies [8, 13], a considerable proportion of cases of macrolide-resistant MP pneumonia might be recategorized as clinical MSMP or MLEP pneumonia. Therefore, the first-line treatment for MP pneumonia can be initially started, even in cases of MRMP pneumonia. Considering the exaggerated immune response in children with MP pneumonia [18], the application of immune-modulators, such as corticosteroids or immunoglobulin, rather than antibiotics, might play more important roles in the management of MP pneumonia, even in some cases of MLEP or MRMP pneumonia [19]. When selecting the proper treatment strategy for MLEP and MRMP pneumonia, consideration of diverse clinical courses, including self-limiting features and combined immune responses in each case, might be more important than the results of in vitro sensitivity tests to macrolide.

This study has some limitations. First, there might have been selection bias due to not including all cases of CAP in Korean children. Second, some patients might have been misclassified into the “no MP pneumonia and no respiratory virus detection” group due to a sampling error, including inappropriate sputum specimens or lack of repetitive follow-up of MP-specific IgM in patients with an initial negative result for MP-specific IgM. Due to the retrospective study design, all techniques of RT-PCR for detection of respiratory viruses and MP and serology for MP were not performed in all participants. In the present study, we did not include CAP caused by typical bacterial pathogens; therefore, we could not identify time-dependent changes in the occurrence of CAP caused by typical bacterial pathogens alone or caused by co-infection of typical bacteria and viruses. However, following the introduction of the pneumococcal conjugate vaccine and the *Haemophilus influenza* type b vaccine, the disease burden due to bacterial pneumonia has been significantly decreased [6, 20]. Therefore, we clinically defined the MSMP, MRMP, and MLEP pneumonia groups solely based on fever duration after initiation of the administration of macrolides, regardless of the results of the in vitro tests for macrolide sensitivity. Because the results of in vitro sensitivity tests to antibiotics do not always correspond with those of in vivo sensitivity tests of the same antibiotics [21] and administration of immune-modulators might play more important roles in the treatment of MP pneumonia in some cases, the application of clinical classifications of MP pneumonia, rather than the results of in vitro macrolide sensitivity tests, might be more helpful in the management of MP pneumonia. This is especially significant in cases of MLEP and MRMP pneumonia, in this era with a high rate of antibiotic-refractory MP.

## Conclusions

We identified the annual and seasonal patterns of causative pathogens in children with CAP requiring hospitalization. Throughout the study period, MP and RSV were the most common causes of CAP, with cyclic peaks in specific seasons. The disease burden due to pediatric CAP requiring hospitalization is largely due to RSV pneumonia, especially in infants and young children. The relatively lower rates of clinical MLEP and MRMP pneumonia, contrary to the known high prevalence of macrolide-refractory MP pneumonia, highlights the appropriate treatment strategies for patients with MP pneumonia. The results of the present study may aid in establishing treatment and management strategies for pediatric CAP and policies to reduce the disease burden by improving public health policies for children with CAP.

## Supplementary information

**Additional file 1: Figure S1.** Time-dependent trends of community-acquired pneumonia due to various respiratory viruses. (A) Influenza. (B) Adenovirus. (C) Human metapneumovirus. (D) Parainfluenza.

## Data Availability

The data used were collected through the routine surveillance systemic of Pneumonia & Respiratory Disease Study Group of Korean Academy of Pediatric Allergy and Respiratory Disease. Dara are available from the authors upon reasonable request and with permission of the Pneumonia & Respiratory Disease Study Group of Korean Academy of Pediatric Allergy and Respiratory Disease.

## Abbreviations

AdV: Adenovirus
BoV: Bocavirus
CAP: Community acquired pneumonia
FLU: Influenza virus
HCoV: Human coronavirus
HMPV: Human metapneumovirus
HRV: Human rhinovirus
MLEP: Macrolide less-effective *Mycoplasma pneumoniae*
MP: *Mycoplasma pneumoniae*
MRMP: Macrolide-refractory *Mycoplasma pneumoniae*
MSMP: Macrolide-sensitive *Mycoplasma pneumoniae*
PIV: Parainfluenza virus
RSV: Respiratory syncytial virus
RT-PCR: Real-time polymerase chain reaction
