# Supplementary figures and images for: Annual and seasonal patterns in etiologies of pediatric community-acquired pneumonia due to respiratory viruses and Mycoplasma pneumoniae requiring hospitalization in South Korea
Source: BMC Infect Dis. 2020 Feb 12;20:132. doi: 10.1186/s12879-020-4810-9 (PMC7017508; doi:10.1186/s12879-020-4810-9)

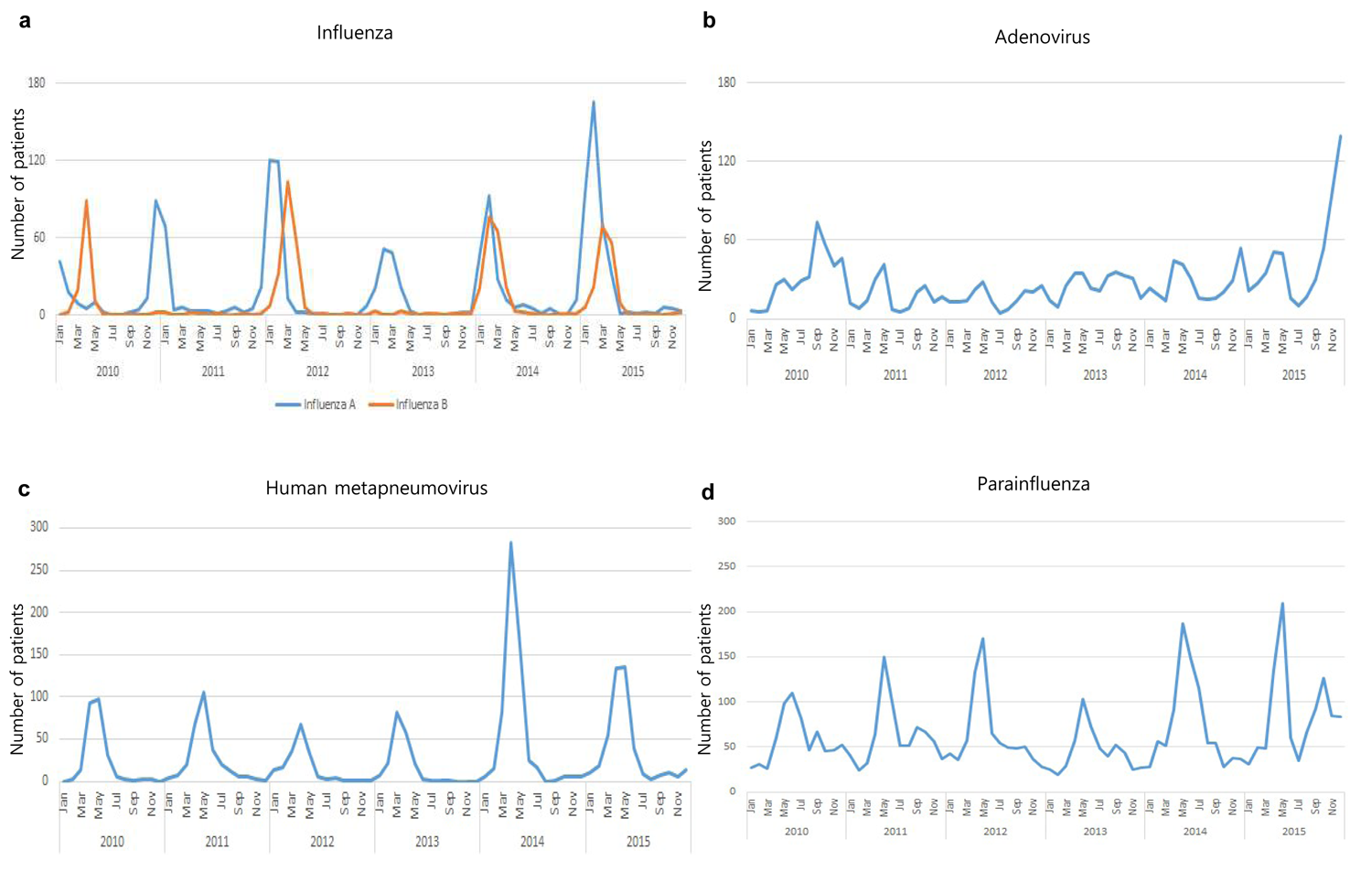

Supplement: Supplementary file 1 — Additional file 1: Figure S1. Time-dependent trends of community-acquired pneumonia due to various respiratory viruses. (A) Influenza. (B) Adenovirus. (C) Human metapneumovirus. (D) Parainfluenza. [file 12879_2020_4810_MOESM1_ESM.tif]
